# Disseminated adenovirus infection following CAR T-cell therapy: Early-onset presentation in a patient with relapsed refractory mantle cell lymphoma

**DOI:** 10.46989/001c.157628

**Published:** 2026-03-30

**Authors:** Anusha Gopalam, Noha Soror, Aqsa Ashraf, Emily Siegrist, Joseph Sassine, Taha Al-Juhaishi

**Affiliations:** 1 University of Oklahoma Stephenson Cancer Center, Oklahoma City, Oklahoma, USA

**Keywords:** Adenovirus viremia, CAR-T therapy, Immunocompromised patients

Chimeric antigen receptor (CAR) T-cell therapy is a highly effective treatment for patients with relapsed/refractory B lymphoid malignancies. However, it carries a significant risk of viral infections, including adenovirus (AdV).^1^ While AdV typically causes a mild infection in immunocompetent individuals, it can result in disseminated, life-threatening disease in immunocompromised patients, manifesting as hemorrhagic cystitis, pneumonia, hepatitis, nephritis, and meningoencephalitis.^2–4^ Mortality in disseminated AdV disease can reach up to 80%.^2,5^

While AdV infection is well-recognized following allogeneic hematopoietic cell transplantation (HCT), limited data exist regarding AdV infection after CAR T-cell therapy.^4,6–8^ Consequently, management strategies have largely been extrapolated from allogeneic HCT guidelines, despite differences in timelines of immune reconstitution.^6,9^ Moreover, treatment options for disseminated AdV infection are limited. Of the available antiviral agents, cidofovir is most used, despite its high risk of nephrotoxicity.^2,3,5^ More data are needed on the clinical course of AdV-infection post CAR T-cell therapy, along with clearer guidelines for antiviral indications and duration.^3,5^

Here, we describe a case of disseminated AdV infection following CAR T-cell therapy in a patient with B-cell lymphoma, and review the current literature on incidence, risk factors, timeline, diagnosis, treatment, and prognosis.

Our case involved a 64-year-old man who presented with gross hematuria with clots, dysuria, and suprapubic discomfort two weeks after receiving CAR T-cell therapy for relapsed mantle cell lymphoma (MCL). He reported significant pain during urination, increased urinary frequency, and passage of blood clots. He remained afebrile, and vital signs were within normal limits.

He had been diagnosed with MCL two years prior to CAR T-cell therapy, after evaluation for a left-sided neck mass and mild dysphagia. Initial treatment included bendamustine, cytarabine, rituximab, and acalabrutinib, but disease progression occurred due to poor treatment adherence. He later developed malignant airway obstruction resulting in hypoxic respiratory failure, vocal cord paralysis, and tracheostomy placement. He received salvage therapy with rituximab, ifosfamide, carboplatin, etoposide, and then pirtobrutinib as a bridge to CAR T-cell therapy.

Prior to CAR T-cell therapy, he received lymphodepleting therapy with fludarabine and cyclophosphamide followed by brexucabtagene autoleucel infusion. On day +3, he developed grade 2 cytokine release syndrome (CRS) with fever and hypoxia, as well as mild Immune effector cell associated-neurotoxicity syndrome (ICANS), requiring ICU admission and treatment with one 800-mg dose of IV tocilizumab, five 100-mg doses of IV anakinra, and twelve 10-mg doses of IV dexamethasone. CRS resolved prior to the onset of urinary symptoms.

On presentation, the initial workup revealed WBC 4.8 x10⁹/L, hemoglobin 10.8 g/dL, platelets 92 x10⁹/L, and creatinine 0.96 mg/dL. Immunological status revealed hypogammaglobulinemia, with IgG level of 301 mg/dL, absolute CD4 T-cell count of 174.8 cells/µL (relative 23%), and absolute CD19 B-cell count of 0 cells/µL. Urinalysis showed >100 Red blood cells (RBC) and 3+ leukocyte esterase. Bladder ultrasound demonstrated irregular mucosal thickening and mobile echogenic material suspicious for clot burden, consistent with hemorrhagic cystitis. AdV viremia was confirmed with a plasma PCR showing 6,700 copies/mL at onset of symptoms, peaking at 11,500 copies/mL. Urine AdV PCR was 2.60 x 10^9^ copies/mL. AdV was also detected on a respiratory PCR panel by nasopharyngeal swab, though he remained asymptomatic. BK viral loads in plasma and urine were not detected.

He was treated with a single 2.5 mg/kg IV dose of cidofovir with concurrent probenecid and hyperhydration; the cidofovir dose was adjusted to his renal function. Urinary symptoms improved rapidly within one week. Renal function and blood counts were monitored daily, and he had a peak creatinine of 1.22 mg/dL (baseline 0.88). He received two additional weekly maintenance doses of cidofovir (2.5 mg/kg IV). Plasma AdV viral load became undetectable (Figure 2), and cidofovir was discontinued. The patient remained asymptomatic and in remission from MCL.

**Figure 1.**
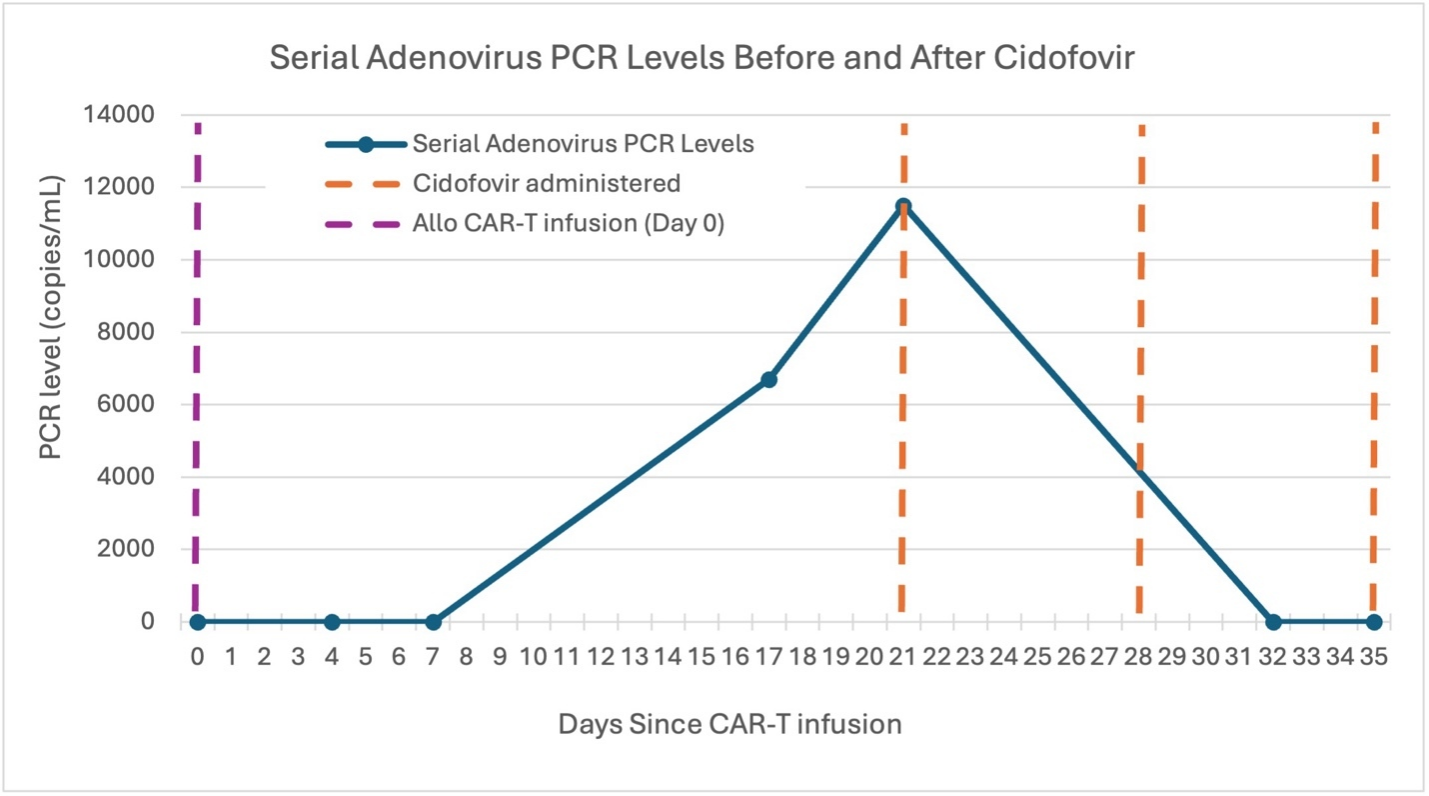
Serial adenovirus (AdV) plasma PCR levels (copies/mL) in our patient following CAR T-cell therapy. Days are plotted since CAR T-cell infusion. Vertical dashed lines indicate allogeneic CAR T-cell infusion (purple) and cidofovir administration (orange).

Disseminated AdV infection remains an uncommon but potentially life-threatening complication following CAR T-cell therapy, with limited data available to guide management. Only a few cases have been reported in the literature (Table 1).^7,10^ Infectious complications are common in the first 90 days after CAR T-cell therapy, when patients are immunosuppressed due to lymphodepleting chemotherapy, cytopenias, B-cell aplasia, and hypogammaglobulinemia.^6,9,11^ AdV is less frequent than bacterial or fungal infections, but may be rapidly fatal when it occurs.^2^ Most reported AdV infections occur 30-60 days post-infusion, during the intermediate phase of immune recovery when T-cell function remains impaired, but early cytopenias have resolved.^1,11–13^

**Table 1.** Summary of published case reports of disseminated adenovirus infection following CAR T-cell therapy in patients with lymphoma, including the case described in this report.

	Case	Khan et al.^7^	Medina et al.^10^
Patient Age/Sex	64 y/o M	70 y/o M	40 y/o M
Malignancy	MCL	DLBCL	DLBCL
CAR T product	Brexucabtagene autoleucel	Axicabtagene ciloleucel	Axicabtagene ciloleucel
Complications	CRS, ICANS	CRS	CRS
Time of onset of infection from CAR T infusion	Day +16 post-infusion	Two months	Two weeks
Presentation	Hemorrhagic cystitis	Hemorrhagic cystitis (co-infection with BK virus)	Hemorrhagic cystitis
Peak PCR viral load	Detected on respiratory panel, urine, and serum Peak urine viral load of 2.60 x 10^9^ copies/mL Peak serum viral load of 11,500 copies/mL	AdV detected in urine and serum BK detected in urine Peak urine viral load of BK of 1,300,000 copies/mL	Detected in urine and serum Peak urine viral load of >20,000,000 copies/mL Peak serum viral load of 259 copies/mL
Treatment	Single dose of IV cidofovir 2.5 mg/kg, followed by 2 maintenance doses, with probenecid	IV cidofovir 1 mg/kg/day, 3x/week, for 3 weeks, with probenecid	Single intravesical 5 mg/kg dose of cidofovir, followed by IV cidofovir 1 mg/kg/day, 3x/week, for 1 week, without probenecid
Outcome	Resolution of symptoms within 1 week of initiation of therapy, remission from MCL	Resolution of hematuria, sustained improvement within 2 weeks of completion of therapy	Discontinued cidofovir after 1 week, no recurrence of HC or viremia, remission from DLBCL

Key features include patient demographics, CAR T product used, clinical complications, timing of AdV onset, presentation, peak PCR viral load, treatment, and outcomes. Data were obtained from published literature (Khan et al.^7^ and Medina et al.^10^), and from chart review of the patient presented in this report.

However, our case developed disseminated AdV infection within weeks post-infusion, significantly earlier than the typical timeline. This early onset suggests that patients with significant preexisting immunosuppression may have a lower threshold for viral reactivation or dissemination than previously appreciated. CRS can further exacerbate immune dysfunction, and immunosuppressive therapies like corticosteroids and tocilizumab used for management of CRS or ICANS can delay T-cell reconstitution and increase infection risk.^6,7,9,11^ Our patient developed AdV infection shortly after CRS treatment, raising suspicion for the link between early immunosuppression, particularly of T-cell mediated immunity, and viral dissemination.

Diagnosis of disseminated AdV is challenging due to its nonspecific presentation that can overlap with other complications. Plasma PCR is the primary diagnostic tool for disseminated AdV; however, there is no recommendation for or data to support routine monitoring in CAR T-cell recipients.^2,12^ The absence of reliable early biomarkers can delay diagnosis and contribute to rapid clinical deterioration and poor outcomes.^4^

Treatment options remain limited. Cidofovir is the most used antiviral agent, but it is associated with variable efficacy and significant nephrotoxicity, especially in patients with renal impairment or concurrent nephrotoxic exposures, and the dosing regimen and treatment duration for this indication is poorly defined.^2,3,5^ Our patient received cidofovir and demonstrated virological improvement. Brincidofovir, the prodrug of cidofovir, is associated with a lower risk of nephrotoxicity; however, the development of its oral formulation was limited by significant gastrointestinal graft-versus-host-disease and its intravenous formulation remains under investigation (NCT04706923).^14,15^

AdV infection following CAR T-cell therapy is a complex and underrecognized complication that poses significant diagnostic and therapeutic challenges. The timing of AdV infection observed in our case, which occurred much earlier than typically described in the literature, underscores the importance of heightened vigilance in the early post-infusion period. While cidofovir remains the primary treatment, its nephrotoxic effects limit its use, and cidofovir dosing regimens are poorly defined. Ongoing research into safer, more effective therapies and strategies for infection prevention and immune reconstitution will be critical to improving outcomes as CAR T-cell therapies continue to expand in use.

## Authors’ Contribution

All authors contributed equally to the following:

Conceptualization

Data curation

Methodology

Project administration

Resources

Supervision

Writing – original draft

Writing – review & editing
